# The impact of ranitidine on monocyte responses in the context of solid tumors

**DOI:** 10.18632/oncotarget.7211

**Published:** 2016-02-05

**Authors:** Ava Vila-Leahey, Dakota Rogers, Jean S. Marshall

**Affiliations:** ^1^ Dalhousie Inflammation Group, Dalhousie University, Halifax, Nova Scotia, Canada; ^2^ Department of Microbiology and Immunology, Dalhousie University, Halifax, Nova Scotia, Canada

**Keywords:** breast cancer, monocyte, MDSC, Immunology and Microbiology Section, Immune response, Immunity

## Abstract

Monocytes and myeloid derived suppressor cells (MDSC) have been implicated on the regulation of tumor growth. Histamine is also important for regulating MDSC responses. Oral administration of the H2 receptor antagonist ranitidine can inhibit breast tumor growth and metastasis. In the current study, we examined the impact of oral ranitidine treatment, at a clinically relevant dose, on multiple murine tumor models. The impact of ranitidine on monocyte responses and the role of CCR2 in ranitidine-induced tumor growth inhibition were also investigated. Oral ranitidine treatment did not reduce tumor growth in the B16-F10 melanoma, LLC1 lung cancer and EL4 thymoma models. However, it consistently reduced E0771 primary tumor growth and metastasis in the 4T1 model. Ranitidine had no impact on E0771 tumor growth in mice deficient in CCR2, where monocyte recruitment to tumors was limited. Analysis of splenic monocytes also revealed an elevated ratio of H2 versus H1 expression from tumor-bearing compared with naïve mice. More detailed examination of the role of ranitidine on monocyte development demonstrated a decrease in monocyte progenitor cells following ranitidine treatment. Taken together, these results reveal that H2 signaling may be a novel target to alter the monocyte population in breast tumor models, and that targeting H2 on monocytes via oral ranitidine treatment impacts effective tumor immunity. Ranitidine is widely used for control of gastrointestinal disorders. The potential role of ranitidine as an adjunct to immunotherapies for breast cancer and the potential impact of H2 antagonists on breast cancer outcomes should be considered.

## INTRODUCTION

Monocyte recruitment is important for tumor progression [1–4]. A subset of monocytes will develop into tumor-associated macrophages; such cells can enhance tumor cell progression, angiogenesis, extravasation, metastasis, and resistance to chemotherapeutics [4–7]. Therefore a potential method to limit tumor progression would be to target monocyte development. Studies in mice have shown that there are alterations in tumor development when monocytes are depleted or their recruitment is inhibited [3, 8–11]. In humans, treatments targeting monocytes are undergoing clinical trials [11, 12].

Monocyte development in the bone marrow of mice is dependent on monocyte colony stimulating factors, such as CSF1 [13, 14]. Hematopoietic stem cells can develop into common myeloid progenitors (CMPs; Lin^−^Thy1^−^IL-7Rα^−^Sca1^−^c-Kit^+^FcγR1^lo^CD34^+^) which have the potential to develop into granulocyte-macrophage progenitors (GMPs; Lin^−^Thy1^−^IL-7Rα^−^Sca1^−^c-Kit^+^FcγR1^hi^CD34^+^) [15]. Subsequently such cells can go on to develop into macrophage and dendritic cell precursors (Lin^−^c-Kit^+^CD115^+^CD135^+^ Ly6C^−^CD11b^−^) [16, 17], and then common monocyte progenitors (Lin^−^c-Kit^+^CD115^+^ CD135^−^Ly6C^+^CD11b^−^) [17]. These common monocyte progenitors may then develop into monocytes [17] which are CD11b^+^Ly6C^hi^ [16, 18]. The Ly6C^hi^ monocytes leave the bone marrow to be part of the peripheral blood monocyte population [16].

Once recruited into the circulation, the Ly6C^hi^ population can differentiate into Ly6C^low^ circulating monocyte population [16, 18], or be recruited to sites of inflammation where they can differentiate into monocyte-derived dendritic cells or macrophages [19]. These inflammatory monocytes are usually CCR2^hi^CX_3_CR1^low^. The Ly6C^low^ population is thought to “patrol” the endothelium and is involved in endothelial repair [20]. These cells are also required for the extravasation and tissue invasion of inflammatory monocytes during infection [21] and usually are CX_3_CR1^hi^. The inflammatory monocyte population is the predominant target for monocyte depletion with the aim of reducing tumor progression.

A subset of monocytes make up part of a group of cells noted as myeloid-derived suppressor cells (MDSCs). MDSCs are a population of immature monocytic and granulocytic cells that have immunosuppressive functions. With cancer progression, there are elevated levels of cytokines such as CSF1-3 and stem cell factor, which leads to abnormal myelopoiesis [22, 23] and an increase in immature myeloid cells in circulation [23], which can develop into MDSCs. Suppressive functions of MDSCs include inhibition of cytotoxic and helper T cell activation and proliferation [24], induction of T regulatory cells [25], reduction of NK cell activity [26], and induction of immunosuppressive macrophage phenotypes [27, 28]. MDSCs have been found to be a barrier to inducing an effective immune response against tumors, even in the context of immunotherapy.

Histamine is increased in concentration within tumors and regulates immunity [29, 30]. Histamine signals through four known histamine receptors (H1-4) which are differentially expressed on all immune cells, including monocytes and MDSCs. Monocytes can express H1, H2, and H4 [31–37], and MDSCs express H1-3 [38–40]. H2 signaling has been implicated in the regulation of monocytes since it enhances CCL2 production and their expression of CCR2, which would enhance monocyte recruitment [41]. H2 signaling can inhibit production of cytokines such as TNF [42] and IL-27 [43] and also induces IL-1β production by monocytes [44]. H2 signaling also inhibits synthesis of reactive oxygen species in monocytes [45–47]. Yang *et al* [38] revealed that histamine signaling, primarily *via* H2 receptors, was important for MDSC function and that lack of HDC caused myeloid cells to remain in an immature state. Another study revealed that cimetidine, an H2 antagonist, inhibited nitric oxide synthesis and arginase I expression in monocytic MDSCs [39, 40], and caused MDSC apoptosis [40]. Histamine has also been shown to be important for inducing proliferation and survival of monocytic MDSCs through H1 and H2 signaling [39]. While functional aspects of MDSCs have been investigated, the impact of H2 signaling on monocyte and MDSC development is poorly understood.

H2 antagonist treatment can inhibit breast cancer development [48]. This is associated with a decrease in monocytes in the spleen and bone marrow. In the current study, we examined a variety of tumors and the impact of ranitidine on their development. Notably, ranitidine did not reduce tumor growth in several non-breast cancer models although it selectively reduced E0771 primary tumor growth and 4T1 metastasis. Using the orthotopic E0771 breast tumor model the impact of H2 antagonists was not observed in CCR2-deficient mice with defective monocyte recruitment. Further analysis revealed a difference in monocyte histamine receptor expression in tumor-bearing compared with naïve mice. Monocyte progenitors were decreased in non-tumor-bearing mice following ranitidine treatment. Populations of monocytes in tumor-bearing mice were also altered in the presence of ranitidine. These results reveal that enhanced tumor immunity in the presence of ranitidine is associated with changes in monocytic cell populations and is CCR2-dependent.

## RESULTS

### Ranitidine does not alter tumor development in the absence of CCR2

In previous studies we demonstrated that ranitidine treatment decreased 4T1 lung metastasis by 61% compared to control mice and reduced the growth of orthotopic primary E0771 breast tumors [48]. The impact of ranitidine treatment on tumor growth was further investigated using a panel of five tumor models; only E0771 primary tumor growth was significantly altered by ranitidine treatment (Table 1). Monocytic MDSCs have been implicated as important for the impact of ranitidine on breast tumor progression. We therefore analyzed the myeloid cell populations in tumor-bearing mice 7 days after tumor cell injection. The percentage of myeloid cell subsets in the spleen were unaltered in LLC1, B16-OVA, and EL4 following ranitidine treatment. The total number of monocytes in ranitidine-treated 4T1 tumor-bearing mice was previously found to be decreased by 46.3% (*p* < 0.005) [48]. There were increased CD11b^+^ myeloid cells in the spleen of ranitidine-treated E0771 tumor-bearing mice, and increased neutrophils in ranitidine-treated 4T1 tumor-bearing mice compared to control mice (Table 2). As ranitidine selectively decreased primary E0771 tumor growth and this was associated with myeloid cell changes we further analyzed the relationship between ranitidine treatment and monocytes in tumor development utilizing this model.

**Table 1:** Final tumor weights of histamine receptor antagonist-treated tumor-bearing mice

	Control		Ranitidine		N = (/group)
	Mean (g)	± SEM	Mean (g)	± SEM	
**B16-OVA**	1.33	0.45	1.74	0.4	7-8
**LLC-1**	0.20	0.05	0.16	0.03	12
**EL4**	0.55	0.08	0.66	0.08	8
**E0771**	0.94	0.19	0.35*	0.10	11-12
**4T1**	0.64	0.07	0.61	0.06	15

* *p* < 0.05, unpaired *t*-test.

**Table 2:** Summary of the splenic myeloid population of histamine receptor antagonist-treated tumor-bearing mice 7 days after tumor cell injection

		% CD11b+ of live		% Ly6C+ of CD11b+		% Ly6G+Ly6Clow of CD11b+		
		Mean	SEM	Mean	SEM	Mean	SEM	N = (/group)
**B16-OVA**	**Control**	10.78	0.82	7.42	0.38	19.5	1.36	14
	**Ranitidine**	12.67	1.64	7.52	0.71	17.37	2.73	14
**LLC-1**	**Control**	10.67	0.51	7.48	0.91	29.39	2.03	9
	**Ranitidine**	13.45	2.67	7.16	0.67	28.38	3.09	9
**EL4**	**Control**	15.35	1.88	6.54	0.88	19.2	2.40	9
	**Ranitidine**	16.3	1.30	6.44	1.04	16.56	1.86	9
**E0771**	**Control**	6.90	0.25	7.79	0.25	21.62	1.42	12
	**Ranitidine**	8.92**	0.65	7.50	0.60	24.44	1.83	12
**4T1**	**Control**	8.26	0.64	7.96	1.00	32.70	1.94	13
	**Ranitidine**	7.63	0.76	6.54	0.68	40.94*	2.83	13

* *p* < 0.05,

** *p* < 0.01, unpaired *t*-test.

**Figure 1:**
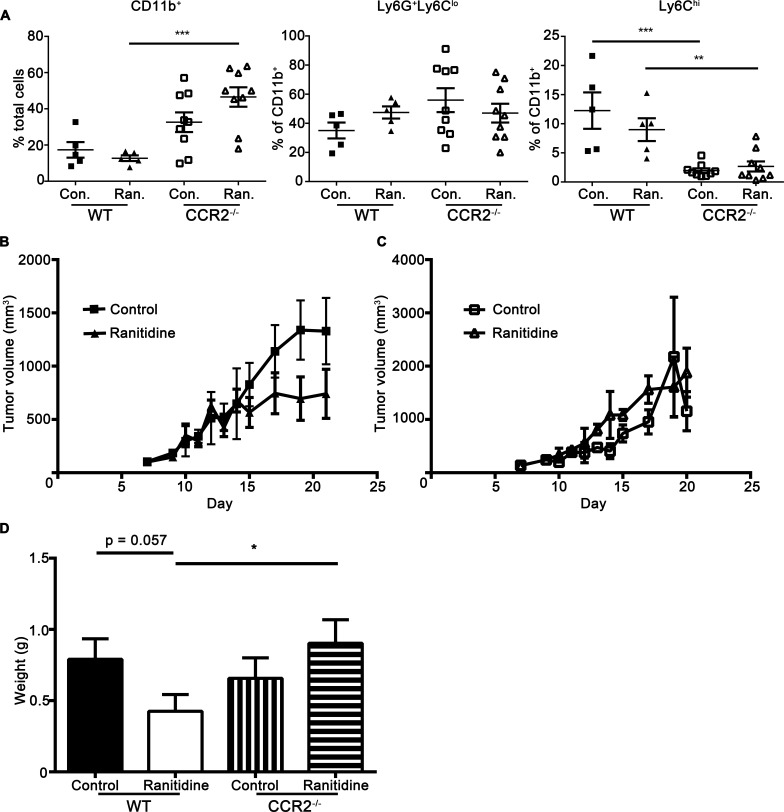
The impact of ranitidine on E0771 tumor progression is associated with changes in circulating monocytes **A.** Composition of blood CD11b^+^ cells, Ly6G^+^Ly6C^low^ granulocytic cells, and Ly6C^hi^ monocytic cells of E0771 tumor-bearing C57BL/6 (WT) and CCR2^−/−^ C57BL/6 mice at day 21. E0771 tumors in C57BL/6 **B.** and CCR2^−/−^ C57BL/6 **C.** mice treated with ranitidine (8 mg/kg) were measured every 2 days starting 7 days post E0771-GFP cell injection. **D.** At day 21, the primary tumor was excised and weighed. Data in **A.** represents individual mice and the line represents mean ± SEM per group. Data points in **B.**-**C.** represents the mean ± SEM tumor volume of 12-20 mice. Data points in **D.** represent final tumor weight of individual mice and line represents the average per group.* *p* < 0.05, **p < 0.01, ***p < 0.001, unpaired *t*-test.

E0771 cells were injected into CCR2^−/−^ C57BL/6 mice. Analysis of blood from these mice showed decreased levels of monocytes in the CCR2 knockout mice compared to wild type mice (2.0 % *vs* 12.3% in CCR2^−/−^ and wildtype mice, respectively). In control C57BL/6 mice, ranitidine caused inhibition of tumor development, starting at approximately day 13 of tumor development. In the CCR2^−/−^ mice, there was no difference in tumor growth or final tumor weight between ranitidine treated and control groups (Figure 1). These results demonstrate a critical role for CCR2 in the mechanism of action of ranitidine and suggest monocytes and/or recruitment of monocytes to the tumor may be important for the impact of ranitidine on tumor progression.

### Analysis of monocytes in E0771-bearing C57BL/6 mice

**Figure 2:**
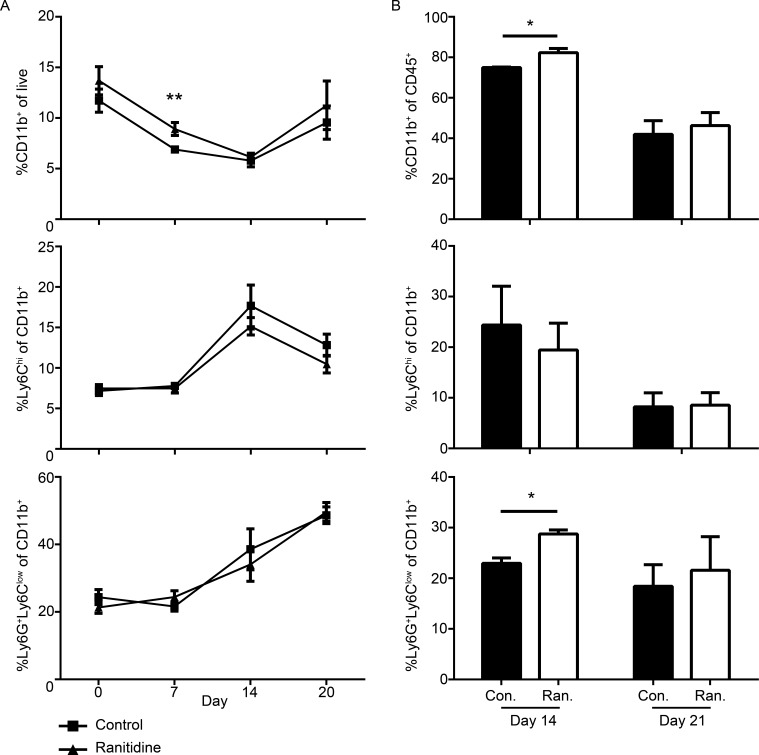
Alterations in myeloid cells at day 14 post E0771 cell injection Composition of CD11b^+^ cells, Ly6G^+^Ly6C^low^ granulocytic cells, and Ly6C^hi^ monocytic cells of splenic cells **A.**, and isolated from the tumor **B.** from E0771 tumor-bearing mice over time. Day 0 in **A.** represent non-tumor-bearing C57BL/6 mice after 8 days of treatment, while the rest represent days after tumor cell injection. Data in **A.**-**B.** represents mean ± SEM of 3-17 mice/group. *p < 0.05, **p < 0.01, unpaired *t*-test.

The nature of the monocyte populations in E0771 tumor-bearing mice with or without ranitidine treatment was further analyzed by flow cytometry. In naïve mice, the monocyte populations were not altered in either the spleen or the bone marrow following ranitidine treatment (Figure 2A). Seven days post tumor cell injection there was a small but significant increase in the overall numbers of myeloid cells in the spleen of ranitidine-treated tumor-bearing mice compared with control tumor-bearing mice that was not due to alterations in identified monocytes or neutrophils (Figure 2B). Spleens and tumors were also analyzed 14 days post tumor cell injection. At day 14, a time point when tumor growth plateaus with ranitidine treatment, an increase in myeloid cells is seen at the tumor site, associated with neutrophil recruitment (Figure 2B). There were no significant differences in the splenic myeloid cell populations at this time point, and no myeloid cell alterations in the spleen or in the tumor at the end point of the experiment (day 20) with ranitidine treatment.

### Ranitidine does not impact circulating monocytes

Circulating monocytes were analyzed during tumor development to see if there were alterations in surface markers. We also analyzed whether there were any differences in CCR2 and CX3CR1 to determine if there were alterations in inflammatory monocyte numbers in circulation in the context of ranitidine treatment. For these studies, mice were treated orally with ranitidine treatment initiated seven days prior to tumor cell injection.

Starting ranitidine treatment one week prior to tumor cell injection caused tumor growth to slow, to an equivalent extent as starting treatment one day prior to tumor cell injection (Supplementary Figure 1). Over the course of the experiment there was an increase in myeloid cells in circulation, but no significant alterations were seen in the total monocytic cells or in inflammatory monocytes in the circulation. However, at the end point of the experiment, there was a significant decrease in monocytes in the spleen in tumor-bearing mice treated with ranitidine compared with control tumor-bearing mice.

### Monocytes modify histamine receptor expression in the presence of a tumor

Histamine receptors are known to be expressed on monocytes [31–37], and the ability of ranitidine and histamine to modulate MDSC function is highly dependent on histamine receptor expression. Therefore we examined the expression of histamine receptors on the subset of monocytes, with characteristics associated with MDSCs, during tumor progression.

**Figure 3:**
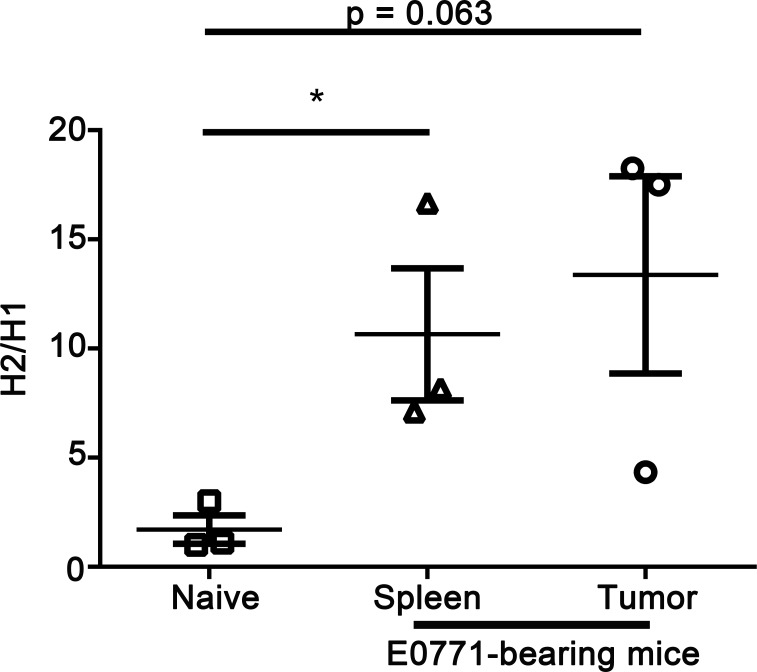
H2 levels are increased compared to H1 in monocytic MDSCs from E0771-bearing mice compared to naïve mice qPCR on CD11b^+^Ly6C^+^CD49d^+^ isolated from E0771 tumors, spleens from E0771-bearing mice, and spleens from naïve C57BL/6 mice was performed for detection of H1 and H2, and a ratio of H2 to H1 expression was calculated. Data points represent individual mice and line represents the mean ± SEM per group. *p < 0.05, unpaired *t*-test.

Haile *et al* [49] describe that monocytic cells expressing CD49d are suppressive, therefore we sorted for CD11b^+^Ly6C^hi^CD49d^+^ monocytic MDSCs and determined H1 or H2 mRNA expression by qPCR. Splenic monocytic MDSCs from tumor-bearing mice had a higher ratio of H2 to H1 compared to monocytic MDSCs from naïve mice (10.6 *vs* 1.7, respectively) (Figure 3). The ratio of H2 to H1 in tumor-associated monocytic MDSCs was also higher compared to naïve mice (13.4 *vs* 1.7, respectively).

### Ranitidine does not alter tumor-associated monocytes

**Figure 4:**
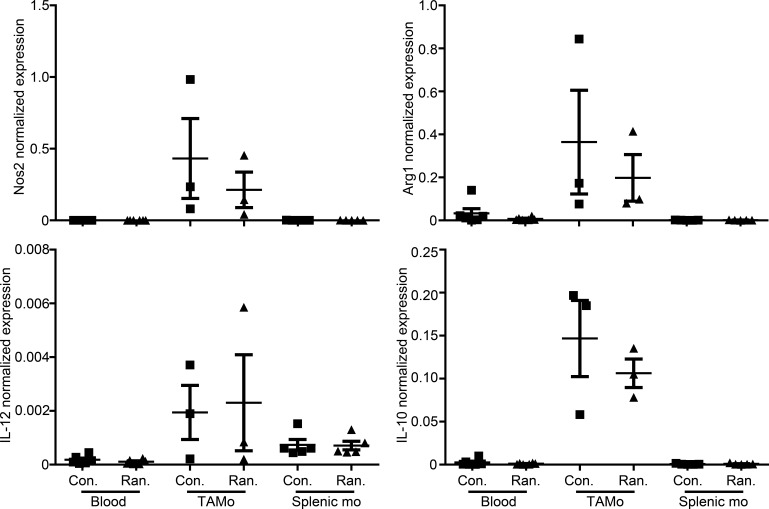
Ranitidine treatment does not alter mediator expression in monocytic MDSCs qPCR on whole blood leukocytes and isolated CD11b^+^Ly6C^+^CD49d^+^ monocytic MDSCs from E0771 tumors (tumor-associated monocytes [TAMo]) and spleens (splenic mo) from E0771-bearing mice 14 days post tumor cell injection were performed for the detection of NOS2, Arg1, IL-12, and IL-10. Data points represent individual mice and line represents the mean ± SEM per group.

To analyze whether ranitidine alters monocytes in the spleen or tumor of E0771 tumor-bearing mice, monocytes were sorted by FACS in a similar manner as previously stated using the markers CD11b^+^Ly6C^hi^CD49d^+^ for monocytic MDSCs, and expression of key mediators was assessed by qPCR. There were no significant alterations in any of the measured mediators between the control and ranitidine-treated groups (Figure 4).

### Analysis of mediators involved in monocyte differentiation and recruitment

**Figure 5:**
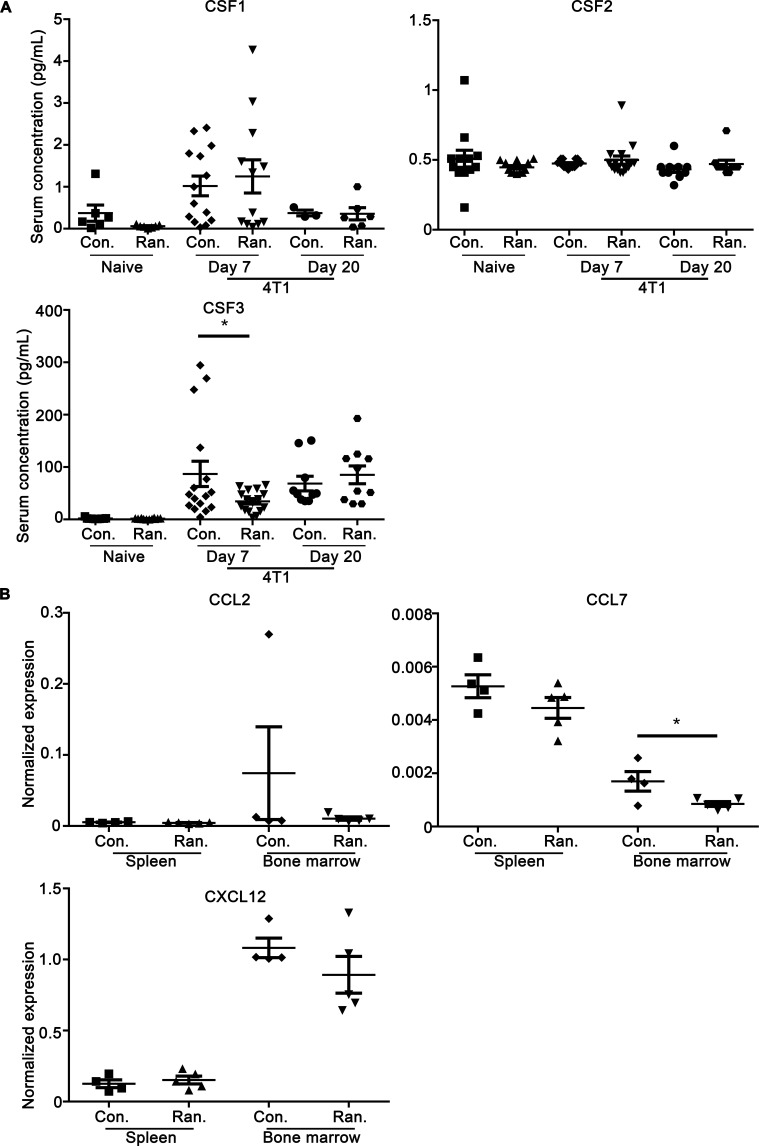
Ranitidine alters CSF3 in 4T1 tumor-bearing BALB/c mice after 7 days **A.** CSF1, CSF2, and CSF3 levels in BALB/c naïve mice, and 4T1 tumor-bearing mice (7 days or 21 days after tumor cell injection) with and without ranitidine treatment was analyzed using Luminex. **B.** qPCR of splenocytes and bone marrow cells was performed for detection of CCL2, CCL7, and CXCL12. Data in **A.**-**B.** represent individual mice and line represents the mean ± SEM per group. *p < 0.05 unpaired *t*-test.

As monocytes are pivotal for tumor development and metastasis and ranitidine was shown to impact monocyte populations most strongly using the metastatic 4T1 tumor model, we utilized this model to further analyze the mechanism of monocyte modulation by ranitidine in tumor-bearing animals. Levels of mRNA expression and presence of mediators in the plasma that can alter monocyte differentiation and recruitment were determined. Analysis of plasma samples from BALB/c mice with and without ranitidine treatment for the presence of colony stimulating factors (CSF1-3) that are involved in myeloid differentiation showed no differences in naïve mice, but in 4T1 tumor-bearing mice there was a significant decrease in CSF3 at day 7 in the ranitidine-treated group, which disappeared after 21 days (Figure 5A). Levels of mRNA expression for chemokines that are important for recruitment of monocytes were also examined. Expression of CCL2, CCL7, and CXCL12 in the spleen and bone marrow in BALB/c mice, treated with ranitidine for 6-9 weeks, was determined. CCL2 mRNA levels were not altered in either area as a result of ranitidine treatment while CCL7 showed a trend towards a decrease in ranitidine-treated animals in the spleen, and significantly decreased in the bone marrow (Figure 5B). CXCL12 trended towards a decrease with ranitidine treatment but this was not statistically significant (Figure 5B).

### Long term ranitidine use alters splenic and bone marrow monocytes and progenitor cells

**Figure 6:**
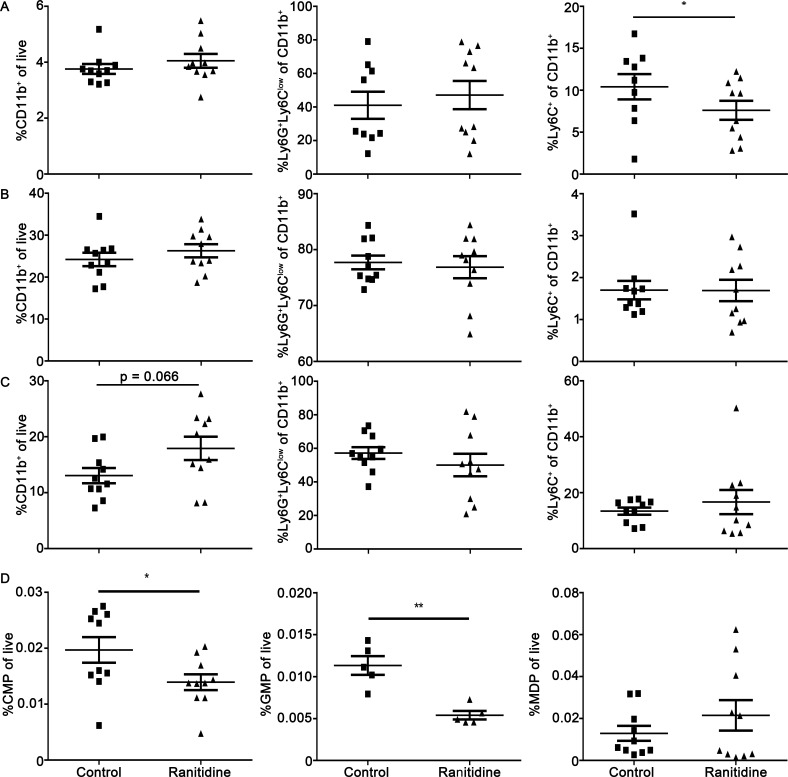
Ranitidine treatment causes a decrease in CMPs and GMPs in naïve BALB/c mice **A.**-**C.** Composition of total CD11b^+^ cells, Ly6G^+^Ly6C^lo^ granulocytic cells, and Ly6C^hi^ monocytic cells in spleen **A.**, bone marrow **B.**, and peripheral blood cells **C.** of non-tumor-bearing mice with and without 6-9 weeks of ranitidine treatment. **D.** Composition of total CMP, GMP, and MDP cells in bone marrow. Data points represent individual mice and line represents the mean ± SEM per group. *p < 0.05, **p < 0.01, unpaired *t*-test.

The impact of ranitidine treatment on monocyte and monocyte-related progenitor cell populations in the bone marrow was also determined in a long term model using non-tumor-bearing animals. The spleen, blood, and bone marrow of BALB/c mice that were treated with ranitidine for 6-9 weeks were examined in comparison with control mice. Long term ranitidine use lead to significantly decreased monocyte populations in the spleen (Figure 6A). Analysis of peripheral blood showed no significant alterations in myeloid cells, although there was a trend toward an increase in myeloid cells in the blood. The numbers of myeloid cells were not decreased in the bone marrow in these animals. When analyzing progenitor cells in the bone marrow, there was a significant decrease in GMPs and CMPs following ranitidine treatment (Figure 6D) but no significant alteration in monocyte progenitors downstream of GMPs. There were also no significant alterations in total splenocyte, bone marrow cell, and peripheral blood cell numbers.

## DISCUSSION

Ranitidine is a widely used drug for the treatment of acid reflux, but also has an impact on immune cells. Although considered to be a safe drug with few side effects, the impact of consistent oral ranitidine on the immune system in a cancer setting has not been fully analyzed. Ranitidine is recommended for the treatment of the gastric side effects associated with chemotherapy [50]. Therefore understanding how clinically-relevant doses of ranitidine may impact the immune system in a tumor-bearing host is important. In our study long term ranitidine treatment was associated with a decrease in splenic monocytes and monocyte progenitors in the bone marrow. In breast tumor models, ranitidine limited tumor growth or metastasis, where altered monocyte populations were also observed. The impact ranitidine had in decreasing E0771 tumor growth was CCR2-dependent and therefore potentially linked to monocyte recruitment. Monocytic MDSCs from E0771 tumor-bearing mice had an increased ratio of H2 to H1 compared to naïve mice. The observed alterations in monocyte populations following ranitidine treatment have implications beyond breast cancer immunity.

After 8 days of ranitidine treatment, there were decreased monocytes in naïve and tumor-bearing BALB/c mice; with long term ranitidine treatment there were significant differences in the monocyte population in the spleen, which were not seen in the bone marrow. Extramedullary hematopoiesis occurs in the spleen to create a monocyte reserve [51]; under steady state conditions monocytes can migrate back into the bone marrow and contribute to the monocyte pool [16]. There is therefore potential for ranitidine to have an impact on splenic progenitor cells in the spleen or in the bone marrow.

In humans, ranitidine treatment-associated myelo-depletion is primarily considered to induce neutropenia [52] while, in mice, our studies suggest that ranitidine-induced myelo-depletion was monocyte specific. A previous study by Byron *et al* [53], showed that H2 signaling pushes bone marrow stem cells from G_0_ to S phase, therefore allowing for stem cell proliferation to occur. Our experiments support these data, but further show that this is specific to CMPs and GMPs. These results suggest that the mechanism of ranitidine-induced decreases in mature monocytes is *via* decreasing the number of monocyte progenitor cells. There is also potential that CMPs and GMPs are decreasing in numbers because they are being mobilized into circulation. Our data showed that ranitidine causes a decrease in CCL7 and a trend towards decreased CXCL12 (Figure 3), and CXCL12 is important for retention of stem cells in the bone marrow [54].

The impact of ranitidine on monocyte progenitors and mature monocytes has clinical implications, although there are very few studies that have directly examined these issues in humans. Decreases in monocyte numbers can further impact multiple different disease states. In a tumor setting, alterations in monocyte numbers and localization can lead to alterations in tumor infiltrate populations, including tumor-associated macrophages [7]. H2 antagonists are often prescribed to patients that are going through chemotherapy, therefore there is potential that H2 antagonist treatment might be beneficial. Alternatively, in patients with chronic inflammatory diseases where monocytes and MDSCs are important for regulating the immune response [55, 56], there is potential to exacerbate disease.

To our knowledge this is the first time differences in histamine receptor expression has been shown in monocytes in a tumor-bearing animal *versus* a naïve animal. High levels of histamine can increase H2 expression [57, 58] and there are elevated levels of histamine in breast cancer patients [29, 30]. H2 signaling is considered to create an immunosuppressive state, including enhancing MDSC survival [39, 40] and inducing mediators such as NOS2 and arginase [39]. H2 antagonists may also impact monocyte survival or the activation of MDSCs. Although MDSCs were isolated from both naïve and tumor-bearing mice, there were differences in receptor expression, suggesting that alterations in receptor expression, that may help promote the survival of MDSCs, may be another mechanism of inducing an immunosuppressive environment in the tumor microenvironment.

The use of CCR2^−/−^ mice compared with wild type mice revealed that the impact ranitidine has on tumor development in the E0771 model of breast cancer is CCR2-dependent. The CCL2-CCR2 axis is critical for recruitment of monocytes to the tumor [7]. Although CCR2 is found on other immune cells, the splenic monocyte and monocyte progenitor data provided supports that the CCR2-dependent effect we observed, is most likely due to monocytes. In humans, there is potential that H2 antagonists can directly impact the accumulation of neutrophils, or that neutrophil numbers are impacted indirectly by altered monocytes, as lack of monocyte recruitment can lead to enhanced neutrophil numbers in a tumor [8]. However, our data strongly implicates monocytes are the key cells in the ranitidine-dependent effect on breast tumor growth and spread.

In conclusion, we show that the impact of ranitidine on tumor development is associated with alterations in the monocyte population and associated progenitor cells. H2 blockade leads to a decrease in monocyte progenitors and alterations in myeloid cell numbers in the tumor. Inhibition of monocyte recruitment, through CCR2 deficiency, prevents the action of ranitidine in reducing tumor growth. These results suggest a mechanism by which H2 blockade can cause a decrease in tumor development when monocyte responses are important for tumor growth. The alteration in H2 expression in monocytic MDSCs suggests that specific blockade of H2 signaling in monocytes and MDSCs could inhibit their development. Overall, these data suggest that ranitidine usage may have effects on monocyte populations with far reaching implications for immune regulation in the context of both breast cancer and other diseases.

## MATERIALS AND METHODS

### Cell lines

Mouse melanoma B16-F10 transduced with ovalbumin (generously provided by Dr. John G. Frelinger and Dr. Edith Lord), mouse breast carcinoma 4T1, mouse lymphoma EL4, mouse lung carcinoma LLC1, and mouse breast adenocarcinoma E0771 (ATCC) transduced with GFP were maintained in a monolayer in Dulbecco's Modified Eagle's Medium (Hyclone) containing 10% fetal bovine serum, and 1% L-glutamine, HEPES, penicillin/streptomycin; for E0771 4 μg/mL of puromycin were added to media for selection of GFP-positive cells, and for B16-F10 500 μg/mL of G418 was added to media for selection of ovalbumin-expressing cells.

### Mice

All mouse experiments were pre-approved by the Dalhousie University Committee on Laboratory Animals. Five week old female BALB/c mice and C57BL/6 mice were purchased from Charles River Laboratories and housed in specific pathogen-free conditions at the Carleton Animal Care Facility at Dalhousie University. CCR2 knockout C57BL/6 mice were bred at the IWK Health Centre animal facility.

### *In vivo* orthotopic breast cancer model

Ranitidine was added to drinking water one day prior to tumor cell injection and was refreshed every other day. 6-8 week old C57BL/6 and CCR2^−/−^ C57BL/6 mice (generously provided by Dr. Thomas Issekutz) were anesthetized and 2 × 10^5^ E0771 cells in 100 μL Matrigel^®^ (Corning) were injected subcutaneously into the mammary fat pad near the fourth nipple. 6-8 week old BALB/c mice were anesthetized and 1 × 10^5^ 4T1 cells in 50 μL PBS were injected subcutaneously into the mammary fat pad near the fourth nipple. The volume of the tumor was determined by caliper measurements every second day using the equation volume = length x width^2^/2. At day 14 or day 21 post injection, the mice were sacrificed and the primary tumor, peripheral blood, and spleen were collected.

For analysis of circulating monocytes, starting one week prior to tumor cell injection and performed weekly, 100 μL peripheral blood was isolated *via* facial vein bleed and processed for flow cytometry.

### Alternate tumor models

A similar experimental layout was used as stated above. 6-8 week old C57BL/6 mice were anesthetized and 2 × 10^5^ LLC1 cells in 50 μL PBS, 1 × 10^5^ B16-OVA cells in 50 μL PBS, or 2 × 10^5^ EL4 cells in 100 μL PBS were injected subcutaneously in the back. At day 7 or day 14-15 (for LLC1 and EL4) or day 20-21 (for B16-OVA), the mice were sacrificed and the primary tumor, peripheral blood, and spleen were collected.

### Flow cytometry

Antibodies: Rat anti-mouse CD11b-fluorescein isothiocyanate (cat. #11-0112, eBioscience), rat anti-mouse Ly6G-biotin (cat. #12760, Biolegend), rat anti-mouse Ly6C-allophycocyanin (APC) (cat. #17-5932, eBioscience), rat anti-mouse CD49d-phycoerythrin (PE) (cat. #12-0492), rat anti-mouse CD62L-PE (cat. #12-0621, eBioscience), rat anti-mouse Ly6C-PE-Cy7 (cat. #25-5932, eBioscience), rat anti-mouse CX_3_CR1-PerCP/Cy5.5 (cat. #149009, BioLegend), rat anti-mouse CCR2-APC (cat. #FAB5538A, R&D Systems). Appropriate isotype matched control antibodies were used in all experiments.

E0771 tumors were digested in the following enzyme cocktail in HBSS: 4.48 U/mL Dispase (cat. #17105, Gibco), 200 μg/mL DNAse I, 10 mM magnesium chloride. The tumors were then pushed through a 100 μM cell strainer and blood was lysed with ACK buffer (0.15 M ammonium chloride [cat. #A4514, Sigma Aldrich], 0.01 M potassium bicarbonate [cat. #P7682, Sigma Aldrich], 0.07 mM EDTA [cat. #15575, Invitrogen]).

Splenocytes, blood, and bone marrow cells were blocked in FACS buffer containing rat serum. Samples were then mixed with primary antibodies for 15 minutes on ice, washed, and mixed with streptavidin PerCP for 20 minutes at 4°C. Following washing, cells were fixed with 1% paraformaldehyde (for acquisition) or resuspended in FACS buffer (for sorting) and acquired/sorted for analysis using a Becton Dickinson FACSAria II. Results were analyzed using FCS express software (De Novo Software).

### qPCR

RNA from cells was isolated using the Qiagen RNA RNeasy Plus Mini kit and QIAamp RNA Blood Mini Kit. Reverse transcription was carried out using the Qiagen Quantitiect Reverse Transcription kit. For qPCR, cDNA was mixed with primers for GAPDH, HPRT, CCL2, CCL7, CXCL12, H1, H2, NOS2, Arg1, IL-10, IL-12 (Quantitect Primer Assay, Qiagen), and Promega GoTaq^®^ qPCR Master Mix. The mixtures were then read in a Stratagene Mx 3000P using the MxPro program, using the following settings: 95° for 5 minutes; 40 cycles of (95°C for 10 seconds, 60°C for 30 seconds); 95° for 1 minute; 55°C for 30 seconds; 95° for 30 seconds. The critical threshold (Ct) of each sample was then obtained and used for normalization compared to the average Ct between GAPDH and HPRT.

### Luminex

25 μL of serum from mice isolated *via* cardiac puncture was used for a ProcartaPlex^TM^ Mouse Basic Kit (eBioscience) to detect CSF1, CSF2, CSF3, IL-6, IL-10, IL-12, and TNF according to manufacturer's instructions

## SUPPLEMENTARY MATERIAL FIGURE


