# Supplementary material for: The impact of ranitidine on monocyte responses in the context of solid tumors
Source: Oncotarget. 2016 Feb 5;7(10):10891–904. doi: 10.18632/oncotarget.7211 (PMC4905447; doi:10.18632/oncotarget.7211)
Supplement: Supplementary file 1 [file oncotarget-07-10891-s001.pdf]

The impact of ranitidine on monocyte responses in the context of solid tumors

Supplementary Material

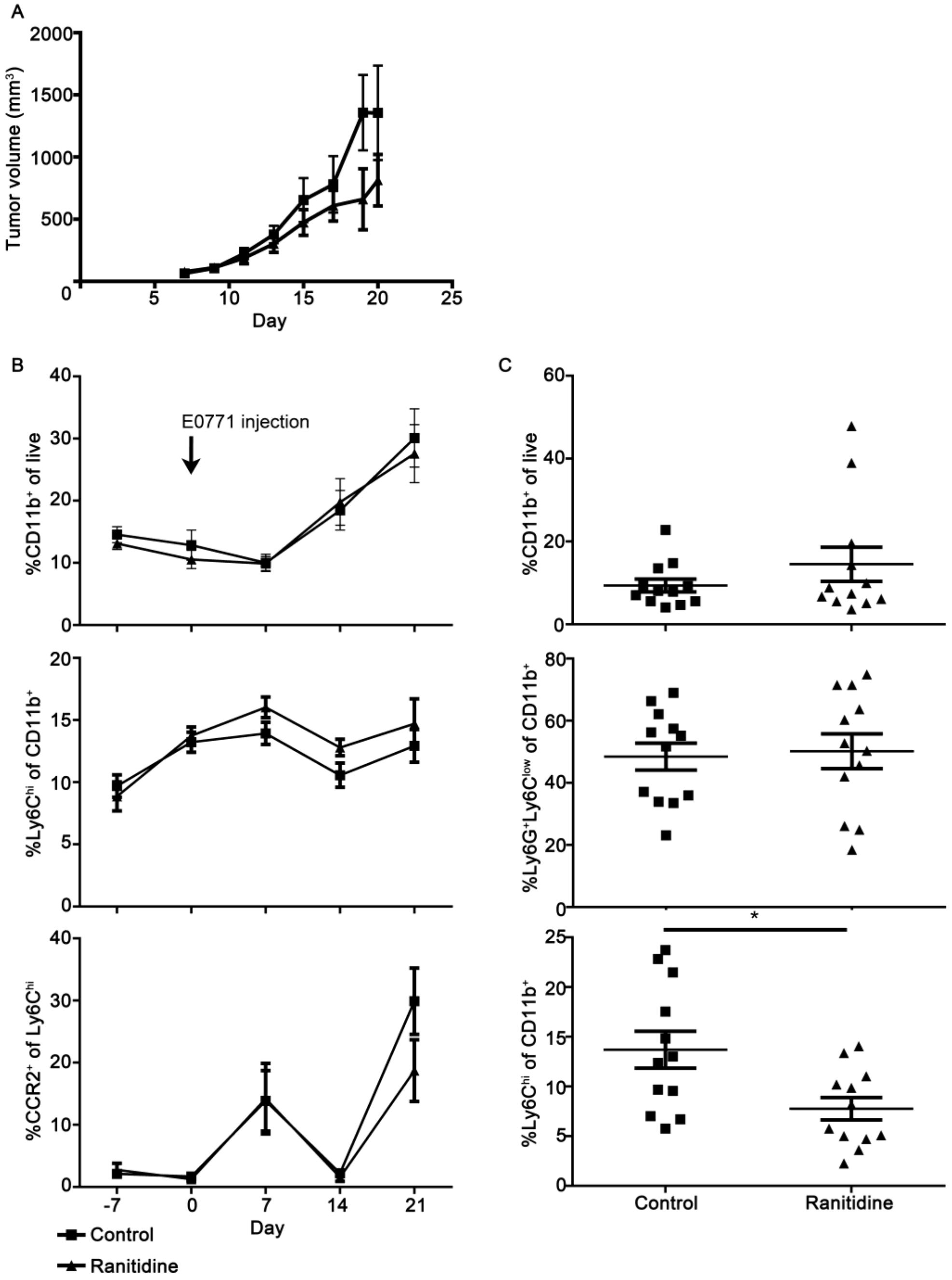

Supplementary Figure 1: Ranitidine treatment, initiated 7 days prior to tumor cell injection, does not impact circulating monocytes but decreases splenic monocytes. (A) E0771-GFP tumors in C57BL/6 mice treated with ranitidine (8 mg/kg) were measured every 2 days starting 7 days after tumor cell injection. (B) Composition of blood $CD11b^+$ cells, $Ly6C^{hi}$ monocytic cells, and $CCR2^+$ inflammatory cells were measured starting 7 days pre-E0771 injection and measured every 7 days. (C) Composition of splenic $CD11b^+$ cells, $Ly6G^+Ly6C^{low}$ granulocytic cells, and $Ly6C^{hi}$ monocytic cells were measured at day 21. Data in (A) represents the mean ± SEM tumor volume of 12 mice. Data points in (B) represents the mean ± SEM percentage of blood cells of 12 mice. Data in (C) represents individual mice and the line represents mean ± SEM per group. *$p<0.05$, unpaired t-test.
